# P38 The gut that leaks

**DOI:** 10.1093/rap/rkac067.038

**Published:** 2022-09-28

**Authors:** Khin Yein, Shivani Gor, Elizabeth Price

**Affiliations:** Great Western Hospital, Swindon, United Kingdom; Great Western Hospital, Swindon, United Kingdom; Great Western Hospital, Swindon, United Kingdom

## Abstract

**Introduction/Background:**

Systemic lupus erythematosus is a chronic inflammatory autoimmune disease which can affect multiple organ systems. Symptoms of intestinal involvement in SLE may vary from common and non-specific such as heartburn, oral ulcers and dysphagia to less common but more serious including abdominal pain due to serositis and intestinal vasculitis.

**Description/Method:**

A 20 year old girl was referred to the gastroenterology team for the management of ascites. Her past medical history included SLE diagnosed aged 10, class IV lupus nephritis, autoimmune haemolytic anaemia secondary to SLE, Haemoglobin H, gastrostomy feeding due to poor growth.

At the time of the transition from paediatric to adult service her SLE was under good control. She was on Prednisolone 6 mg on alternate days, Mycophenolate (MMF) 750 mg BD, Septrin 240 mg OD and Enalapril 2.5 mg OD. After the transition, MMF was reduced to 500 mg OD. The rest were unchanged. Serum total protein was 57 (60-80), serum albumin 32 (35-50). Weight was 41.2 kg.

Ten months after the transition, she presented with bilateral leg swelling and subsequently tense ascites over 3 weeks. Serum total protein reduced to 31, albumin 13.

24 hour urine total protein was 0.15 (0-0.1), urine volume 1.7 L. Serum creatinine 20 µmol/L and eGFR >90. Urine PCR was 16 mg/mmol.

Total protein in ascites drain fluid was <20 g/L, albumin <10 g/L, glucose 5.7 mol/L. There were no malignant cells and no growth on enrichment culture.

She was referred to the dietitians who advised that her oral intake was adequate.

There was no evidence of heart failure on echocardiogram.

Small bowel meal showed oedematous bowel wall but no abnormalities in terminal ileum. Coeliac serology was negative.

CT abdomen and pelvis showed normal appearances to the liver, hepatic and portal veins; no features of Budd-Chiari syndrome; normal size kidneys; incidental finding of non-occlusive right internal jugular thrombus and moderate lymphadenopathy in mesenteric and retroperitoneal regions.

She developed swelling in the right calf. Doppler ultrasound confirmed major DVT with occlusive thrombus extending to distal superficial femoral veins. Clots in inferior vena cava and portal veins were excluded.

**Discussion/Results:**

Duodenal biopsy confirmed villous oedema and dilated lymphatics consistent with changes seen in protein losing enteropathy. She was referred to Rheumatology. Prednisolone was increased to 15 mg. The response was rapid with reduction in ascites. MMF was increased to maximum tolerable dose 750 mg AM and 500 mg PM.

Hb had been persistently low around 50 during this illness. She had longstanding hypocomplementaemia. C3 dropped to 0.37 (baseline 0.6). C4 remained low around 0.04. IgG was 5.9 (baseline >7). Serology showed DsDNA was 5 (normal range 0-15), positive ANA, trace positive RNP and negative Rheumatoid factor and APL antibodies. CRP was 10.

Abdominal lymphadenopathy was felt to be due reactive changes due to active lupus.

With increased steroids and immunosuppressants, she improved significantly both clinically and biochemically within a few weeks. Hb rose to over 90, serum albumin > 25 and serum protein above 50 by the end of 3 months after treatment escalation.

Warfarin was stopped upon completion of 6 month anticoagulation.

On repeat CT, previous abdominal lymphadenopathy had resolved completely. Splenomegaly measuring 17.6 cm was noted. This was discussed in haematology MDT and thought to be due to the haemoglobinopathy and haemolysis.

Haemolysis continued to be a challenge. She remained on relatively high dose steroids varying between 20 mg and 10 mg over the years. Vitamin D was prescribed. Weight was very low but stable just above 38 kg.

Protein Losing Enteropathy (PLE) is rare but recognised in SLE. It typically occurs in young women with clinically severe disease with multi organ involvement. Clinical presentation is characterised by the onset of profound oedema, hypoalbuminaemia in the absence of nephrotic range proteinuria. Severe diarrhoea is present in 50% of the patients. It is important to investigate and exclude other causes of hypoalbuminaemia (e.g cardiac failure, liver diseases, malnutrition) and PLE.

**Key learning points/Conclusion:**

PLE can be the initial manifestation of SLE.

Lupus nephritis and PLE can be present successively or simultaneously in a lupus patient.

Stringent diagnostic evaluation is required to exclude other mimicking conditions.

Multidisciplinary team involvement is crucial in the management of patients with multi-organ involvement.

PLE associated with SLE usually responds well to corticosteroids. However additional immunosuppressant(s) are generally required to treat other SLE symptoms as well as to help with the steroid reduction regime.

As well as treating the underlying disease, advice from dietitians is important to improve and maximise the nutritional status of these patients.

A clinically significant hyper-coagulable state is reportedly attributed to PLE associated with SLE. Therefore, VTE prophylaxis especially in hospitalised patients is recommended. Moreover, clinicians should have high index of suspicion for VTE in these patients with multiple risk factors to arrange early appropriate investigations and treatment plan.

